# Electroacupuncture Alleviates Visceral Hypersensitivity in IBS-D Rats by Inhibiting EGCs Activity through Regulating BDNF/TrkB Signaling Pathway

**DOI:** 10.1155/2022/2497430

**Published:** 2022-02-14

**Authors:** Ying Zhao, Hui-ling Jiang, Yu Shi, Wei Zhang, Lei-xiao Zhang, Yu-jun Hou, Zuo-qin Yang, Bao-yu He, Fan-rong Liang, Qian-hua Zheng

**Affiliations:** ^1^Acupuncture and Tuina School of Chengdu University of Traditional Chinese Medicine, Chengdu, Sichuan 610075, China; ^2^Department of Chongqing Beibei Traditional Chinese Medical Hospital, Chongqing, China; ^3^Department of Traditional Chinese Medicine, The People's Hospital of Shifang, Shifang, China; ^4^Department of Integrated Traditional and Western Medicine, West China Hospital, Sichuan University, Chengdu 610041, China; ^5^Department of Acupuncture and Moxibustion, Chengdu Pidu District Hospital of Traditional Chinese Medicine, Chengdu, China

## Abstract

**Objective:**

To determine whether electroacupuncture (EA) could alleviate visceral hypersensitivity in diarrhea-predominant irritable bowel syndrome (IBS-D) rats by inhibiting EGCs activity via the BDNF/TrkB signaling pathway.

**Methods:**

Sprague Dawley rats were randomly divided to a control group (*n* = 8) and a model preparation group (*n* = 32), which received Senna solution by gavage and CUMS (chronic unpredictable mild stress) for 14 consecutive days and was further divided to a Model group, an EA group (only electroacupuncture), an EA + TrkB agonist group (electroacupuncture and TrkB), and an EA + DMSO group (electroacupuncture and DMSO, *n* = 8 for each). Rats in the three EA groups were acupunctured at ST25, ST36, and LR3 for 20 min every day for 14 days. Abdominal withdrawal reflex (AWR) was used to quantify visceral sensitivity; reverse transcription polymerase chain reaction (RT-PCR) and double immunofluorescent staining were used to detect the colocalized expression of GFAP/BDNF and GFAP/TrkB. Western Blot (WB) was used to detect the expression of PLC and SP in the colon. Flow cytometry was used to detect the expression of Ca^2+^.

**Results:**

EA effectively alleviated visceral hypersensitivity in IBS-D rats (*P* < 0.05). Compared to the control group, the expression of BDNF, TrkB, PLC, SP, and Ca^2+^ and the colocalized expression of GFAP/BDNF and GFAP/TrkB increased in the Model group (*P* < 0.05), while all these parameters decreased in the EA group following EA intervention (*P* < 0.05). In addition, no significant difference was found between the EA + TrkB agonist group and the control group (*P* > 0.05).

**Conclusions:**

EA alleviates visceral hypersensitivity of IBS-D rats possibly by inhibiting the activity of EGCs through the BDNF/TrkB-PLC-Ca^2+^ signaling pathway in the colon.

## 1. Introduction

Irritable bowel syndrome (IBS) is a chronic functional gastrointestinal disorder with persistent or recurrent episodes of intestinal disorder [1]. It is characterized by abdominal pain, abdominal distension, altered bowel habits, and/or character of stool that severely affect the quality of patients' life [2]. A wide spectrum of clinical manifestations and a lack of a specific diagnostic basis [2] make it difficult to treat. Although medication, nutritional support, and psychotherapy are currently in use, few drugs are available for effective treatment [3]. Acupuncture therapy has received increasing attention, as numerous clinical studies have shown that it is safe and effective for the treatment of IBS. For instance, a recent trial showed that acupuncture was more effective than PEG 4000 or pinaverium bromide for the treatment of IBS and the sustained effect of acupuncture was up to 12 weeks in a large sample cohort [4]. In addition, electroacupuncture (EA) had been shown to alleviate abdominal pain in IBS-D patients [5]. However, the mode of action of EA for ISD-D treatment remains unclear.

Extensive studies had shown that an altered visceral sensitivity is associated with the development of IBS [1]. Enteric glial cells (EGCs) play a critical role in supporting and nourishing the enteric nervous system and the development of visceral hypersensitivity in IBS [6, 7], suggesting EGCs as a potential therapeutic target for gastrointestinal disease [8]. Indeed, alterations in EGCs structure and function are associated with IBS pathogenesis [9]. For instance, intestinal EGCs in IBS patients exhibit an activated state, which releases SP substances [9] that continuously excite intestinal sensory neurons and maintain intestinal visceral hypersensitivity. EGCs can be activated through Brain-derived Neurotrophic Factor (BDNF)-related signaling pathways. With a large amount of BDNF being present in the intestinal mucosa of IBS patients, BDNF has been shown to be involved in the production and maintenance of pain in IBS [10]. It has also been shown that BDNF specifically binds to and thus activates the submucosal EGCs surface tyrosine kinase receptor B (TrkB) in the intestinal mucosa [9], which in turn activates the phospholipase C (PLC)-inositol triphosphate (IP3) [11] signaling pathway. Then, the activated signaling pathway upregulates intracellular Ca^2+^ levels with the rapid increase of extracellular Ca^2+^ levels, eventually leading to the activation of EGCs [12, 13].

Accordingly, acupuncture had been shown to alleviate visceral hypersensitive symptoms by reducing serum BDNF level in IBS-D patients [14]. Interestingly, it was found that EA could exert analgesic effects by modulating spinal microglia and spinal astrocytes activation [15, 16]. Moreover, the similarity of EGCs to central nervous system (CNS) astrocytes has been demonstrated. Thus, these studies provided strong evidence on the molecular basis of EA-included alleviation of IBS-D symptoms.

**Figure 1:**
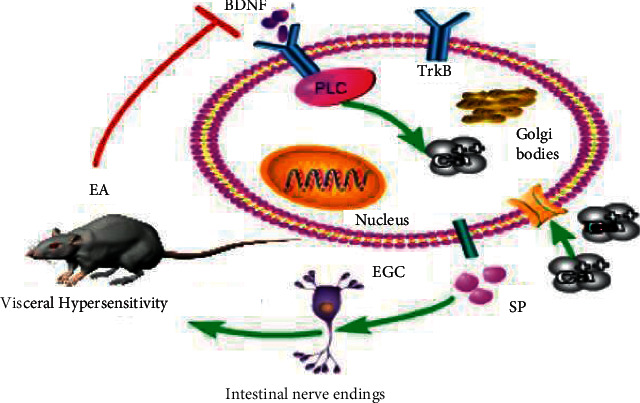
Schematic diagram of the mechanism of acupuncture treatment of IBS in rats. Brain-Derived Neurotrophic Factor (BDNF) specifically binds to and thus activates the enteric glial cells (EGCs) surface tyrosine kinase receptor B (TrkB), which in turn activates the phospholipase C (PLC) signaling pathway. Then, the activated signaling pathway upregulates intracellular Ca^2+^ levels with the rapid increase of extracellular Ca^2+^ levels, eventually leading to the activation of EGCs.

In this study, we speculated that EGCs are a possible target for acupuncture treatment of IBS-D and that BDNF signaling pathway is the key pathway of EGCs activation. Specifically, we hypothesized that EA regulates the activation of EGCs cells by downregulating the BDNF/TrkB pathway, thereby alleviating visceral hypersensitivity in IBS-D rats. To test this hypothesis, we used an IBS-D rat model (Model group) and treated these rats with EA only (EA group), EA with TrkB (EA + TrkB agonist group), or EA with DMSO (EA + DMSO group). Analyses on several parameters including SP levels, BDNF and TrKB protein abundances, and PLC/Ca^2+^ pathways were performed to test our hypothesis (Figure 1).

## 2. Materials and Methods

### 2.1. Animals

A total of 40 female Sprague Dawley rats (7-week-old and weighing 180–220 g) were obtained from Chengdu Dasuo Experimental Animals Co. (Chengdu, China). Animals were maintained in the Key Laboratory of Sichuan Province, China, where the temperature was kept at 18–24°C, with 50–70% humidity and a 12 h/12 h light/dark cycle. Animals were allowed to eat and drink freely. This experiment has been approved by the animal ethics committee of Chengdu University of Traditional Chinese Medicine (approval no. 2019–06), and all the disposal of animals conforms to the National Guideline for the Care and Use of Laboratory Animals.

### 2.2. Rat Model of IBS-D

An IBS-D rat model was established by gavage and CUMS for 14 consecutive days as previously described [17]. Briefly, 32 rats were randomly selected for one of the following CUMS types [18, 19]: 24 h in solitary (one cage), restraint for 2 h, crowding for 24 h (more than 6 in one cage), swimming at 45°C for 5 min, tail clamping for 20 min, or oscillation for 1 h (240 Hz). Each rat received discontinuous CUMS at least twice. Half an hour after each CUMS, rats were given 0.3 g/mL of Senna solution by gavage (10 mL/kg). Abdominal withdrawal reflex (AWR) was used to measure the visceral hypersensitivity. Successfully modeled rats were randomly divided into a Model group, an EA group, an EA + TrkB agonist group, and an EA + DMSO group, with 8 rats in each group. The remaining rats were assigned as a Control group, which was given distilled water by gavage at 10 mL/kg daily.

### 2.3. EA Treatment

Electroacupuncture was performed in rats after meal. Rats in the EA group, EA + TrkB agonist group, and EA + DMSO group received EA for 20 min once per day for 14 days. After being immobilized on a self-made fixator and disinfection of the skin area at the acupoints with 75% alcohol, the rats were treated with EA. The acupoints were ST25 (*Tianshu*, located in the abdomen; the middle side of the umbilicus is opened about 5 mm), ST36 (*Zusanli*, the posterolateral side of the knee joint, about 5 mm under the fibula head), and LR3 (*Taichong*, located at the tibial side of the second toe of the foot, the posterior depression of the metatarsal bone). Stainless steel acupuncture needles (Huatuo, Suzhou Medical Supplies Co., Ltd., Φ0.16 × 13 mm) were inserted into the three acupoints at a depth of 1-2 mm and connected to an EA apparatus (HANS-200A, Nanjing, China). The lateral/dilatational waves were set to a frequency of 2/15 Hz and an intensity of 1.5 mA. Rats in the EA + TrkB agonist group were intraperitoneally injected with a TrkB receptor agonist 7,8-dihydroxyflavone (MedChemExpress, USA) one hour before EA. The 7,8-dihydroxyflavone (7,8-DHF) was dissolved in 10% DMSO administered at a dose of 5 mg/kg through intraperitoneal injection. And the EA + DMSO group was injected with the same dose of DMSO solution, while the control group was only bound on fixator for 20 minutes without EA intervention.

### 2.4. Abdominal Withdrawal Reflex (AWR) Score

AWR was assessed after 24 h fasting as previously described [20]. A balloon of 5 cm in length and a catheter of 4 mm in diameter were used to make a colorectal tension balloon, and the catheter was connected to a sphygmomanometer via a three-way tube. Following anesthesia and being transferred to an acrylic fixed box (18 × 7 × 6 cm) in which the rats cannot turn around, a colorectal dilatation balloon was inserted to the descending colon and a pressure of 20, 40, 60, or 80 mmHg was applied for colorectal dilatation (CRD). AWD scores, blindly evaluated by two trained researchers who did not know the groups' information, were used to assess the degree of visceral sensitivity during rectal dilation in rats [20, 21] with the following scale: AWR0: no response to CRD; AWR1: slight head rotation; AWR2: abdominal muscle contraction; AWR3: abdomen raised; and AWR4: body arched and pelvis raised. After each 20-second CRD, AWR scores were measured three times, and an average value was recorded as the AWR score.

### 2.5. Sample Collection

After EA intervention, rats were intraperitoneally injected with pentobarbital sodium (40 mg/kg). Following anesthesia, the abdominal cavity was opened and the colon tissue was collected. The colon tissues were washed with 0.9% saline and then placed in 4% paraformaldehyde for immunofluorescence detection, 3% glutaraldehyde for transmission electron microscopy detection, and liquid nitrogen at −196°C for WB and RT-PCR detection. After the experiment, rats were killed by cervical dislocation.

### 2.6. Histological Assessment

Colon tissues were fixed in a 10% neutral formaldehyde, dehydrated with 100% alcohol in an automatic dehydrator, embedded in paraffin, and stained with hematoxylin and eosin (H&E). After sectioning, the colon tissue was observed under a light microscope (Jinan Tangier Electronics Co., LTD, China).

### 2.7. Western Blot (WB)

The protein abundance of PLC and SP was quantified by WB in colon tissues. First, total proteins were extracted in RIPA lysis buffer (Beyotime, China) and quantified with the BCA protein quantification kit (Beyotime, China). Samples were separated by electrophoresis, transferred to a PVDF membrane (Sigma-Aldrich, USA), and blocked with 5% skimmed milk. The membrane was incubated with primary antibodies (anti-PLC antibody, 1 : 1000 dilution, rabbit clonal antibody, Abcam, UK; anti-SP antibody, 1 : 1000 dilution, rabbit clonal antibody, Affinity, USA; anti-*β*-actin antibody, 1 : 100000 dilution, rabbit clonal antibody, ABclonal, China). After washing, the membrane was incubated with a secondary antibody (Biotinylated Goat anti-rabbit IgG (H + L), 1 : 5000 dilution, Abcam, UK). Relative expression of the target protein was obtained by normalization against actin.

### 2.8. Real-Time PCR (RT-PCR)

RT-PCR was used to quantify *BDNF* and *TrkB* expression. First, colon tissues were ground in liquid nitrogen for RNA extraction. For each sample, 50–100 mg tissue was extracted with 1 mL of RNA Trizol Reagent (Hefei Bomei Biotechnology Co., Ltd., China). The total RNA was used to synthesize cDNA. Primers (Shanghai SANGON Biotech Co., Ltd., China) are shown in Table 1. PCR reactions were performed on a real-time fluorescence quantitative instrument (QuantStudio TM3, Thermofisher, USA) with a total of 45 cycles. In this study, the relative expression levels of *BDNF* mRNA and *TrkB* mRNA were calculated by 2^−△△CT^.

### 2.9. Immunofluorescence

Double immunofluorescence labeling was performed to determine the expression patterns of GFAP-BDNF and GFAP-TrkB complexes in colon. Tissue was fixed and dehydrated as described above. After being embedded using BMJ-III, samples were sliced by a rotary slicer (Leica-2016, Germany). Slices were immersed in 0.01 M citrate buffer (pH 6.0), heated in a microwave oven at medium-high heat until boiling with an interval of 5 min. Samples were first rinsed with PBS and then blocked with 10% serum (Zhejiang Tianhang Biotechnology Co., Ltd., China) at room temperature for 30 min. Primary antibodies (anti-GFAP, mouse monoclonal antibody, concentration: 1 : 100, Abcam, UK; anti-TrkB, Rabbit polyclonal antibody, 1 : 100, Affinity Biosciences, OH, USA; anti-BDNF, rabbit polyclonal antibody, 1 : 100, Bioss, China) were added to the samples separately. After rinsing the samples with PBS for 30 min at 37°C, a secondary antibody (FITC labeled goat anti-rabbit IgG, ServiceBio, China; Cy3-labeled goat anti-mouse IgG, ServiceBio, China) was added. The samples were dripped with nuclear dye, washed with PBS, and sealed with an antifluorescence attenuation sealing agent. A fluorescence scanning microscope camera system (Jinan Tangier Electronics Co., LTD, China) was used for image acquisition.

### 2.10. Flow Cytometry (FCM)

The content of Ca^2+^ was measured by FCM. Colon tissues were washed with PBS at 4°C and ground. The resulting suspension was extracted, sieved, and centrifuged for 5 minutes to obtain cell precipitations. Cells were resuspended in 500 *µ*L of diluted Fluo 3-AM (Sigma-Aldrich, USA), followed by washing twice with 500 *μ*L of PBS and reloading 300 *μ*L of Fluo 3-AM. Finally, Ca^2+^ was detected and analyzed by a Cytoflex flow analyzer (Beckman, USA).

### 2.11. Statistical Analysis

Statistical analysis was performed using SPSS21.0. After normal test and homogeneity analysis of variance, multiple groups comparison and one-way analysis of variance were used. Post hoc test was performed for pair comparison with the least significant difference (LSD) method. In cases where there were no variances among groups, Tamhane's T2 test was used. *P* < 0.05 and *P* < 0.01 were considered as statistically significant and extremely significant, respectively.

## 3. Results

### 3.1. Effect of EA on Visceral Hypersensitivity

**Figure 2:**
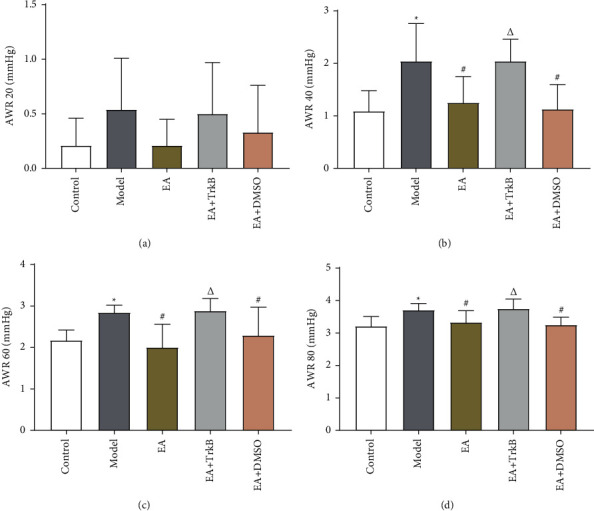
AWR score and SP expression and effect of EA on visceral hypersensitivity. (a) AWR scores with a CRD of 20 mmHg; (b) AWR scores with a CRD of 40 mmHg; (c) AWR scores with a CRD of 60 mmHg; (d) AWR scores with a CRD of 80 mmHg;  ^*∗*^*P* < 0.05, versus the blank control group; ^#^*P* < 0.05 and ^Δ^*P* < 0.05, versus the model group.

AWR score is an important index to evaluate visceral hypersensitivity. As shown in Figure 2(a), there was no significant difference in the AWR score of rats among groups with a CRD of 20 mmHg (*P* > 0.05). As the CRD increased to 40, 60, and 80 mmHg, AWR scores of IBS-D rats were significantly increased compared to the control group (*P* < 0.05). Importantly, the AWR score of IBS-D rats showed a significant decrease after EA treatment (*P* < 0.05), while there was no significant change in the EA + TrkB agonist group (*P* > 0.05).

### 3.2. Histological Assessment

**Figure 3:**
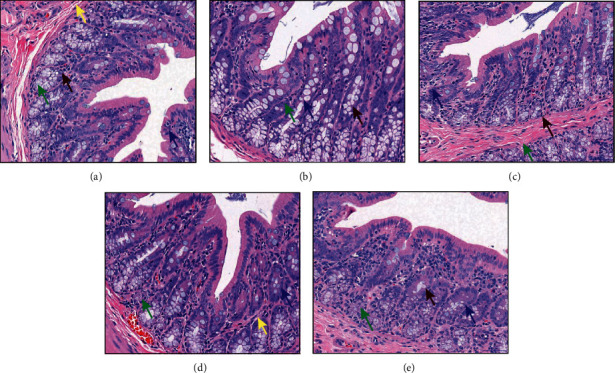
Histological assessment of colon tissues. (a) The control group; (b) the Model group; (c) the EA group; (d) the EA + TrkB agonist group; (e) the EA + DMSO group. Images were taken under a light microscope (×400); goblet cell (**↑**), lymphocytes (**↑**), eosinophils (**↑**), and neutrophils (**↑**).

Histological assessment showed that the colon tissue structure of the control group was complete and normal (Figure 3(a)). By contrast, the Model group showed stratification in the colonic tissue without hyperplasia or ulceration (Figure 3(b)). For the EA group, the tissue was completely stratified (Figure 3(c)). For the EA + TrkB agonist group, the tissue was clear without any obvious degeneration and necrosis (Figure 3(d)). Lastly, the colon of the EA + DMSO group was similar to that of the control group (Figure 3(e)). In addition, the mucosa, submucosa, muscle layer, and outer membrane structure of colon tissue were clear for all groups without any obvious pathological changes.

### 3.3. EGCs Inactivation in Analgesia Effect of EA in IBS-D

#### 3.3.1. Effect of EA on the Structure and Morphology of EGCs

**Figure 4:**
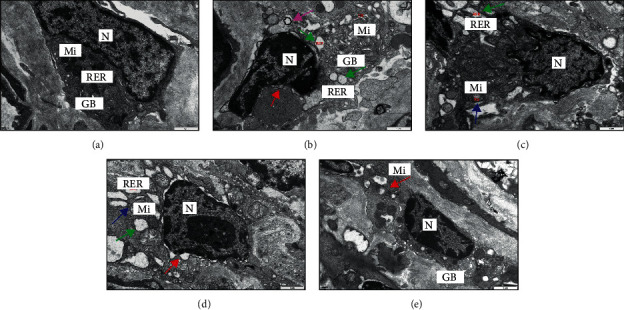
Structure and morphology of EGCs. (a) EGCs of the blank control group; (b) EGCs of the Model group; (c) EGCs of the EA group; (d) EGCs of the EA + TrkB agonist group; (e) EGCs of the EA + DMSO group. Images of EGCs were obtained under TEM (×20000). N, nucleus; Mi, mitochondria; RER, rough endoplasmic reticulum; and GB, Golgi apparatus; widened perinuclear space (**↑**), mitochondria slightly swollen (**↑**), dilated rough endoplasmic reticulum (**↑**), and autophagy (**↑**).

Normal EGCs morphology and structure were observed for the control group with major organelles observed (Figure 4(a)). By contrast, the Model group showed nuclear chromatin edge concentration, widened perinuclear space, more abundant Golgi bodies, and expansion and cystic characters for most of the rough endoplasmic reticulum (rER, Figure 4(b)). For the EA group, a small number of mitochondria in the EGCs cytoplasm showed mild swelling, and a large number of dilated, cystic rER were observed (Figure 4(c)). Although EA promoted EGCs morphologic repair, the effect was not observed in the EA + TrkB agonist group, in which EGCs showed mitochondrial swelling, crest fracture, dissolution, and even disappearance (Figure 4(d)). In the EA + DMSO group, a small number of mitochondria were slightly swollen, and a small part of rER expanded into cystic forms (Figure 4(e)).

#### 3.3.2. Effects of EA on SP

**Figure 5:**
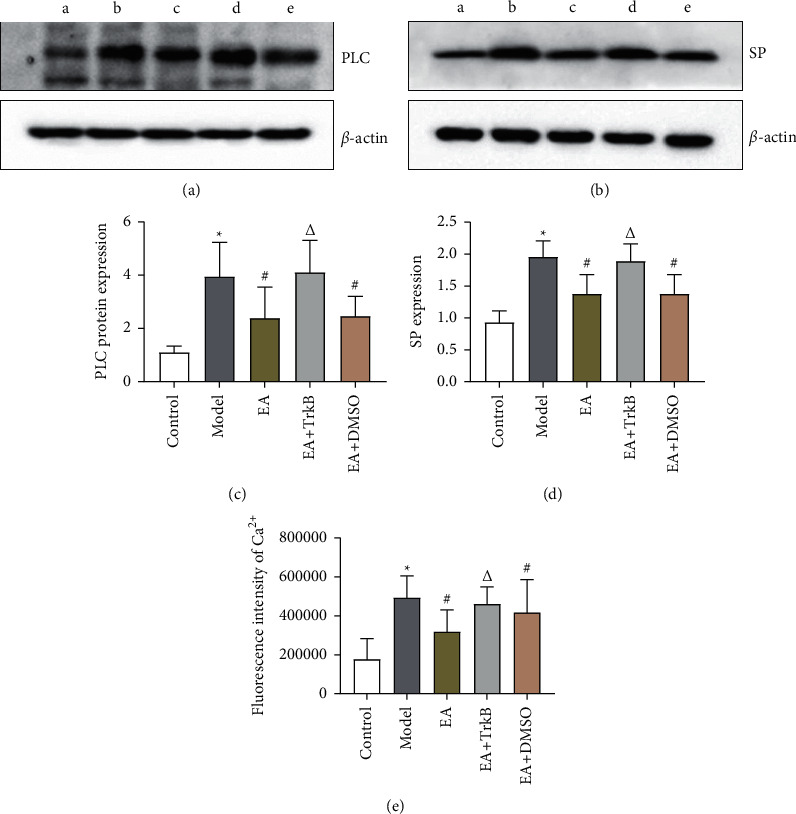
(a–d) The expression of PLC and SP in the colon of each group of rats (a: control group, b: Model group, c: EA group, d: EA + TrkB agonist group, and e: EA + DMSO group). (e) Mean fluorescence intensity of Ca^2+^ in the colon.  ^*∗*^*P* < 0.01, versus the blank control group; ^#^*P* < 0.05 and ^Δ^*P* > 0.05, versus the model group.

After modeling, the colon SP content in IBS-D rats increased significantly (*P* < 0.01, Figure 5(d)). Compared to the Model group, EA resulted in a significant decrease in SP content (*P* < 0.01). As expected, there was no significant change in the EA + TrkB agonist group (*P* > 0.05).

### 3.4. Effect of EA on BDNF/TrkB Signaling Pathway

#### 3.4.1. mRNA Expression of BDNF and TrkB in the Colon

**Figure 6:**
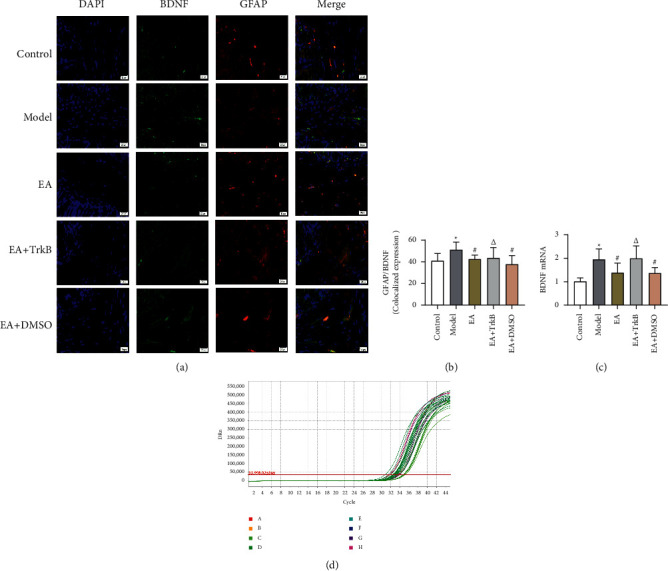
(a) GFAP and BDNF fluorescence in different groups. Green indicates BDNF, red indicates GFAP, and blue indicates DAPI-stained nucleus. (b) Colocalization of GFAP and BDNF. (c) mRNA expression of BDNF in the colon.  ^*∗*^*P* < 0.05, versus the blank control group; ^#^*P* < 0.05 and ^Δ^*P* > 0.05, versus the Model group.

**Figure 7:**
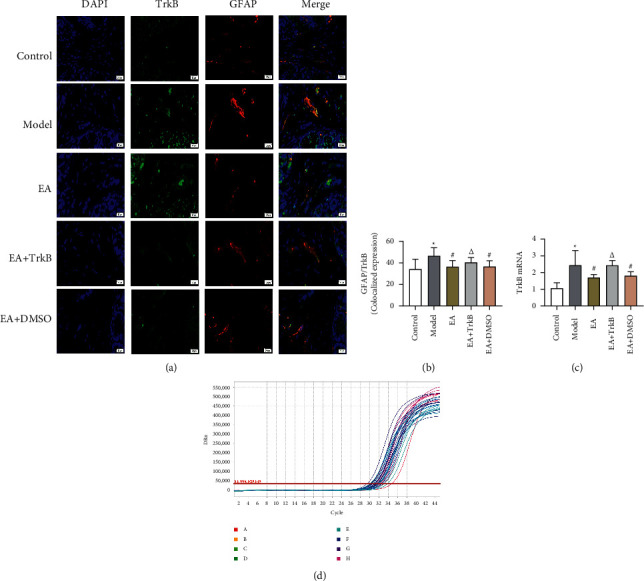
(a) GFAP and TrkB fluorescence in different groups. Green indicates TrkB, red indicates GFAP, and blue indicates DAPI-stained nucleus. (b) Colocalization of GFAP and TrkB. (c) mRNA expression of TrkB mRNA in the colon.  ^*∗*^*P* < 0.05, versus the blank control group; ^#^*P* < 0.05 and ^Δ^*P* > 0.05, versus the Model group.

Senna solution by gavage and CUMS resulted in an increase in the expression of *BDNF* and *TrkB* compared to the control group (Figures 6(c) and 7(c)). In addition, EA stimulation led to a significant decrease in the expression of both genes. Moreover, there was no significant difference between the Model group and the EA + TrkB agonist group.

#### 3.4.2. TrkB-Mediated PLC/Ca^2+^ Signaling Pathway in EGCs Was Activated by EA Stimulation

To further determine whether the upregulation of the PLC-Ca^2+^ pathway was mediated by TrkB, the TrkB agonist 7,8-dihydroxyflavone was used prior to the quantification of PLC protein and Ca^2+^ in colon. Our data showed that the protein abundance of PLC increased after modeling (Figure 5(b)). Compared to the Model group, EA intervention led to a significant decrease of PLC protein abundance in the EA and EA + DMSO groups, but not in the EA + TrkB agonist group. A similar trend was found for Ca^2+^ (Figure 5(e)), where modeling caused an increase in Ca^2+^ content and EA restored the level.

#### 3.4.3. Colocalization of GFAP and BDNF and of GFAP and TrkB in the Colon

To explore whether BDNF in IBS-D rat colon specifically binds to TrkB receptor on EGCs and determine selective inactivation of TrkB in colon by EA, a double-label immunofluorescence assay was performed to assess the colocalization between BDNF and GFAP, as well as between TrkB and GFAP. Our data showed a significant increase in colocalized expression of GFAP and BDNF after modeling (Figure 6(a)). Compared to the Model group, a significant decrease was observed in the EA group and the EA + DMSO group (*P* < 0.05), while no significant change was found in the EA + TrkB agonist group (Figure 6). Similar results are also observed in Figure 7, where the fluorescence intensities for GFAP (red) and BDNF (green) increased in the Model group compared to the control group. In addition, EA led to a decrease in the fluorescence intensity compared to the Model group.

Compared to the control group, EGCs in the Model group showed a strong colocalization with TrkB and exhibited high TrkB fluorescence intensity (Figure 7(b)). Significantly, EA led to a decrease in the colocalized expression of GFAP and TrkB, as evidenced by the observation that EA at ST25, ST36, and LR3 was sufficient to inhibit the expression of TrkB in colon (*P* < 0.05). In addition, there was no significant change in the EA + TrkB agonist group (*P* > 0.05). In line with the GFAP/BDNF results, GFAP/TrkB showed an increase in the Model group, which led to a decrease in the fluorescence intensity compared to the EA group (Figure 7).

## 4. Discussion

The clinical symptoms of IBS are mostly manifested as abdominal pain and bloating, which are typical features of visceral hypersensitivity and an important clinical marker to distinguish IBS from other functional gastrointestinal diseases [22]. A dysregulation in the communication between the gut and brain has also been confirmed for IBS [23]. The cerebral nervous system and the central nervous system are known to share many similarities [24]. While many studies focused on central nervous system such as the central [25] and spinal cord [26], few had explored the enteric nervous system. Mounting evidence has shown that IBS is associated with an increased excitability of sensory neurons in the gut, manifested as hypersensitivity of intestinal receptors to various stimuli and hyperalgesia and allodynia [27]. EA alleviates visceral hypersensitivity in IBS at multiple levels by regulating intestinal dynamics [28], visceral receptor sensitivity [20], intestinal flora [29], and the brain-gut axis [30, 31]. Therefore, this study explored the peripheral mechanism of EA in IBS-D rats. We found that EA resulted in downregulation of the BDNF/TrkB signaling pathway, decreased EGC activity, and lower AWR scores.

In this study, we quantified visceral hypersensitivity using AWR scores, an important index for intestinal sensitivity [32], and found a significant increase with higher CRD pressure in the Model group. The fact that no pathological changes were found in the colon of the Model rats validated the establishment of the model and further confirmed that IBS-D is a functional gastrointestinal disease [33]. More importantly, EA stimulation caused a significant drop in the visceral hypersensitivity of the IBS-D model rats, consistent with a previous report [20]. In addition, a higher CRD pressure was correlated with an enhanced alleviation effect. Furthermore, the alleviation on visceral hypersensitivity by EA was abolished in the EA + TrkB agonist group, suggesting that the modulation of visceral hypersensitivity by acupuncture may be mediated through TrkB receptors.

Glial cells and nerve cells constitute the nervous system, and enteric glial cells (EGCs) belong to the glial cells distributed in the periphery. In the intestine, EGCs are mainly distributed in the submucosal enteric ganglia and enteric myenteric plexus of the enteric nervous system, and GFAP is a biomarker for the activation of EGCs. Under physiological states, EGCs play key roles in maintaining intestinal homeostasis, regulating the intestinal epithelial barrier, and promoting the development of the nervous system [34, 35]. Under pathological states, such as in IBS-D, EGCs can be abnormally activated and secrete substances such as SP to regulate the excitability of intestinal sensory neurons and maintain the visceral hypersensitivity of IBS-D [7, 9]. Previous studies have shown that EGC activation is closely associated with visceral hypersensitivity in IBS [6, 7, 36].

Due to the critical role of EGC in IBS-D visceral hypersensitivity, we observed the morphological and structural changes of submucosal EGCs in the colonic mucosa by using TEM. Compared to the control group, the EA group showed ER expansion, perinuclear gap, and slight swelling of the mitochondria. We thus speculate that the ultrastructure of EGCs in the IBS-D state is altered, and EA may reverse the alteration to some extent. EGCs are also known to secrete SP to stimulate intestinal primary neurons and maintain visceral hypersensitivity. Consistent with previous studies [37], we found that colonic SP substance expression was significantly increased in IBS-D rats and significantly decreased after EA. In addition, our immunofluorescence results showed that GFAP in the Model group was significantly increased compared to the control group, suggesting that the EGCs were in an activated state. On the other hand, EGC inhibitors can alleviate visceral hypersensitivity in IBS rats [9]. Our results showed that EA may alleviate visceral hypersensitivity of IBS-D rats by inhibiting EGC activity as a significant decrease of GFAP was found in the EA and EA + DMSO groups.

Previous studies of IBS-D have shown an increase in the intestinal BDNF, which binds to the TrkB receptor on the submucosal EGC surface of the intestine and activates the PLC-IP3 signaling pathway, triggering a dramatic increase in the intracellular Ca^2+^ level of EGCs and contributing to EGC hyperactivation [9–11, 37, 38]. In line with this, we found that colonic BDNF and TrkB receptors were significantly increased in IBS-D rats. It had been suggested that the increased BDNF in the pathological state of IBS-D specifically binds to the TrkB receptor on the surface of EGCs and activates EGCs. This was assessed by immunofluorescence double staining, in which GFAP was included as a marker of EGCs activation [39]. Our results showed an increase in the colocalized expression of GFAP/BDNF and GFAP/TrkB in IBS-D rats compared to the control group. In addition, we found an increase in PLC protein abundance and Ca^2+^ level along with the activation of the BDNF/TrkB pathway, consistent with previous reports.

Compared to the Model group, EA resulted in a decrease in the colocalized expression of GFAP/BDNF and GFAP/TrkB. Additionally, the levels of colonic BDNF and TrkB receptors, PLC protein, and Ca^2+^ were also decreased. Thus, it is possible that EA reduces PLC and Ca^2+^ content, inhibits EGCs activity, and alleviates visceral hypersensitivity of IBS-D rats by inhibiting the binding of BDNF and TrkB receptors on EGCs. To confirm the mode of action, we injected a TrkB agonist intraperitoneally into IBS-D rats before EA. As expected, no difference was found compared to the Model group with regard to all parameters measured (colocalized expression of GFAP/BDNF and GFAP/TrkB, colonic PLC protein abundance, and Ca^2+^ level). Because DMSO was used as the solvent for the EA + TrkB agonist group, the EA + DMSO group was also included as a control. Importantly, the EA + DMSO indeed showed significant changes for all parameters quantified here. Thus, we conclude that the EA effect was attributable to TrkB receptor but not to DMSO. Taken together, EA alleviated visceral hypersensitivity in IBS-D rats by modulating the BDNF/TrkB-PLC-Ca^2+^ signaling pathway.

## 5. Conclusions

EA improves visceral hypersensitivity of IBS-D rats possibly by inhibiting the activity of EGCs through the BDNF/TrkB-PLC-Ca^2+^ signaling pathway in the colon.

## Figures and Tables

**Table 1:** PCR primers.

Contents	Forward	Reverse
BDNF	GAGCTTTGTGGACCCCTGAGTTC	CCGTGGACGTTTGCTTCTTTCATG
TrkB	GGTCTATGCGTGGTGTGTGTTG	ATGTCTCGCCAACTTGAGCAGAAG
*β*-Actin	GAAGATCAGATTGCTCC	TACTCCTGCTTGCTGATCCA

## Data Availability

All data included in this study are available upon request to the corresponding author.
